# Redox Impact on Bacterial Macromolecule: A Promising Avenue for Discovery and Development of Novel Antibacterials

**DOI:** 10.3390/biom12111545

**Published:** 2022-10-22

**Authors:** Jamiu Olaseni Aribisala, Saheed Sabiu

**Affiliations:** Department of Biotechnology and Food Science, Faculty of Applied Sciences, Durban University of Technology, P.O. Box 1334, Durban 4000, South Africa

**Keywords:** antibiotic resistance, antioxidants, bacterial lethality, oxidative stress, reactive oxygen species

## Abstract

Antibiotic resistance in bacteria has remained a serious public health concern, resulting in substantial deaths and morbidity each year. Factors such as mutation and abuse of currently available antibiotics have contributed to the bulk of the menace. Hence, the introduction and implementation of new therapeutic strategies are imperative. Of these strategies, data supporting the role of reactive oxygen species (ROS) in bacterial lethality are intriguing, with several antimicrobials, including antibiotics such as fluoroquinolones, β-lactams, and aminoglycosides, as well as natural plant compounds, being remarkably implicated. Following treatment with ROS-inducing antimicrobials, ROS such as O_2_^•−^, ^•^OH, and H_2_O_2_ generated in bacteria, which the organism is unable to detoxify, damage cellular macromolecules such as proteins, lipids, and nucleic acids and results in cell death. Despite the unique mechanism of action of ROS-inducing antibacterials and significant studies on ROS-mediated means of bacterial killing, the field remains a topical one, with contradicting viewpoints that require frequent review. Here, we appraised the antibacterial agents (antibiotics, natural and synthetic compounds) implicated in ROS generation and the safety concerns associated with their usage. Further, background information on the sources and types of ROS in bacteria, the mechanism of bacterial lethality via oxidative stress, as well as viewpoints on the ROS hypothesis undermining and solidifying this concept are discussed.

## 1. Introduction

Antimicrobial resistance has evolved as one of the most severe public health challenges of the 21st century, threatening efficient prevention and treatment of microbial infections that are no longer sensitive to most conventional antibiotics [1,2]. Over the years, multidrug-resistant microorganisms (MRM) have developed a varying degree of resistance to each new antibiotic released into the market, with various accompanying dire consequences [3,4]. Yearly, about 700,000 deaths occur worldwide due to infections caused by MRM, and this number could increase to 10 million by the year 2050, depending on resistance evolution patterns and, more importantly, the status of available, effective therapeutic alternatives [5,6,7]. The estimated associated cost of infections caused by MRM is an annual global GDP of 3.8%, culminating in 28.3 million people being subjected to extreme poverty globally [6,7]. Unfortunately, the misuse and abuse of antibiotics, which are majorly implicated in the increased emergence of antibiotic resistance, are still on the rise [1,3,6,7]. Moreover, the inadequate measures for effective prevention and control of microbial infections in developing countries, as well as the increased failure rate in the discovery and development of new therapeutics, have further aggravated the problem of antimicrobial resistance [1,3,6]. Hence, more success in the discovery of new and more potent therapeutics would significantly and undoubtedly help in the fight against antimicrobial resistance [8]. Of the viable strategies in the discovery and development of therapeutics, the contributions of reactive oxygen species (ROS) to the antimicrobial arsenal of antibiotics or antibacterial agents have been proficiently demonstrated and continue to attract research interests [9,10,11,12].

Generally, living organisms depend on oxygen for cellular aerobic respiration and metabolism [13]. However, the existence of oxygen in the form of free radicals may trigger cellular oxidative stress, with possible dire consequences in many organisms [10,14]. During aerobic respiration, ROS are generated in small concentrations, but cell defense mechanisms generally detoxify them without causing damage to cells [11,15]. However, excessive levels of ROS generated via redox reaction or as a result of antibiotic treatment may overcome the cellular defense mechanisms, resulting in oxidative stress. During oxidative stress, small quantities of ROS that cells have limited appropriate defenses against may quickly become lethal, particularly to bacteria [9,10,13]. This concept has long been employed in antiseptics and disinfectants but has not found extensive usage as therapeutics due to ROS’ perceived toxicity and unacceptable damage to the host tissue [8,15]. However, evidence supporting their role in therapeutic lethality has been strengthened in recent times, and novel means of producing ROS have been identified, allowing their therapeutic use to be investigated for topical and systemic infections [8,10,11,16]. The revelation that bactericidal antimicrobials such as fluoroquinolones, β-lactam, and aminoglycoside kill bacteria through the induction of oxidative stress [17,18,19], combined with the knowledge of how generated ROS are utilized by host defense cells (e.g., neutrophils) in pathogen elimination, has sparked renewed interest in the appreciation of ROS and oxidative stress as a novel antibacterial therapeutic strategy.

ROS-inducing agents are finding practical usage as therapeutics. For instance, Surgihoney (produced in the UK by Healing Honey International), a genetically modified kind of honey with wound-healing properties, owes its potent antibacterial actions to high levels of cellular ROS (in the form of H_2_O_2_) as its primary means of antibacterial action [8]. However, future implementation and widespread usage of more novel ROS-inducing antimicrobials (whose primary means of antibacterial action is based on ROS generation) would require insight into the nature of the ROS involved in antimicrobial activity and safety concerns associated with their use [8]. This is necessary, as ROS differ in their toxicity, and host cells have limited defense against ROS such as ^•^OH [8,16]. Moreover, though several studies have implicated the involvement of ROS in the lethality of antibiotics and some natural therapeutic agents [8,16,17,18,19], various other studies citing limitations to this hypothesis have surfaced over the years [20,21,22]. This controversial nature of the field has caught the attention of more current studies and continues to culminate in novel findings. Thus, this study forms part of the ongoing efforts to provide a more balanced insight into the purview of oxidative stress in bacterial lethality.

Previous studies attempting to review ROS-induced antibacterial agents have focused mostly on ROS-inducing antibiotics, with only a few studies on ROS-inducing plant-derived natural and synthetic compounds. Moreover, in recent times, new studies implicating ROS in the antibacterial activity of several novel plant-derived natural and synthetic compounds have surfaced, and this necessitates an up-to-date appraisal. Hence, this study presents information on ROS-induced plant-derived natural (most especially phenolics) and synthetic compounds. Further, as previous reviews have focused on studies implicating ROS in the killing of all microorganisms, this study gathered only information reported on bacteria in an attempt to understand the unique mechanism and type of ROS associated with the killing of bacteria. Equally, information on non-toxicity issues of some ROS-inducing antibacterial agents on human tissues and cells was reviewed and presented for the first time in this study. Background information on the sources and types of ROS generated and antioxidant defense mechanisms in bacteria, the mechanism of bacterial lethality via oxidative stress induction, as well as viewpoints on hypotheses undermining and solidifying the antibacterial potential of ROS, are also discussed in the study. Although this was undertaken with the view to having a better understanding of divergent perspectives on the concept of ROS-mediated bacterial lethality, it is hoped that opinions from this study will guide future studies seeking to understand the role of oxidative stress in the antibacterial potential of synthetic and natural plant-derived therapeutics.

## 2. Materials and Methods

**Figure 1:**
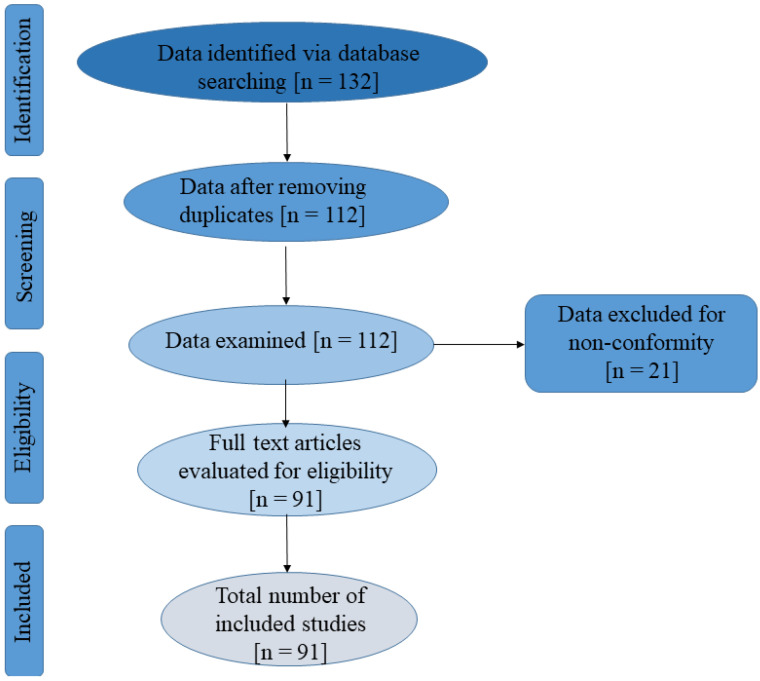
PRISMA flowchart of the literature screened, included, and evaluated for this study.

The Preferred Reporting Items for Systematic Reviews and Meta-Analyses (PRISMA) protocol [23] was followed for the search. Information was gathered between the years 2015 and 2022 from major scientific databases (Google Scholar, Scopus, Science Direct, Web of Science, PubMed, Springer, and BioMed Central) using journals as well as books and/or chapters. Reactive oxygen species and/or oxidative stress were searched and cross-referenced with terminologies such as bacterial and plant interaction, natural plant-derived compounds, phenolics, synthetic compounds, and safety concerns. Following the removal of duplicates, eighteen research articles not within the scope of the study (e.g., studies implicating ROS in the killing of other microorganisms different from bacteria and studies not written in English) were excluded from the studies examined. Ninety-one scientific papers that provided relevant information within the scope of the review were used in the review, and Figure 1 depicts the PRISMA flowchart for this investigation.

### 2.1. Background Information on ROS Sources, Types, Generation, and Impact on Bacterial Macromolecules

#### 2.1.1. Sources and Types of ROS in Bacteria

Aerobic respiration remains the major endogenous source of ROS in bacteria. Respiration in bacteria occurs mostly in the cytoplasm, during which molecular oxygen (O_2_) is transformed into water, carbon dioxide, and energy (in the form of adenosine triphosphate (ATP)) [24]. However, during this process, partial reductions of oxygen species often occur, resulting in superoxide anion (O_2_^•−^) being released from electron transport chains, which are catalyzed into hydrogen peroxide (H_2_O_2_) [15,25,26]. During subsequent reactions, hydroxyl radicals (^•^OH) and singlet oxygen (^1^O_2_) are frequently formed (Table 1) [25,26]. Another endogenous source of ROS in bacteria includes antimicrobial treatment [24]. Endogenous ROS accumulation in bacteria via antibiotic treatment is drug-specific [27]. Antibiotics such as fluoroquinolones, β-lactams, and aminoglycosides, as well as several natural agents, have been demonstrated to increase the rate of respiration in bacteria following treatment, leading to subsequent high endogenous ROS generation [17,18,19]. In addition, some studies have suggested that the primary antibiotics target damage following treatment with antimicrobials, which may trigger mechanisms such as the envelope stress response and programmed cell death, resulting in ROS buildup in bacteria [24,27,28]. Furthermore, the redox cycling potential of some antibacterials (such as nitroaromatic and phenolics) in the bacterial cell have been shown to increase endogenous superoxide anions. These agents in the presence of oxygen in bacterial cells deplete NADH to derive their toxicity.

Exogenous sources of ROS in bacteria occur as a result of host–pathogen interaction, which results in bacteria being attacked by ROS of the host cell [29]. Due to ROS’ ability to act as a signaling molecule in the host, ROS are capable of inciting host defense (e.g., phagosomes) against several bacteria [30] during infection. In this case, specialized phagocytes such as neutrophils and macrophages engulf pathogens into a membrane-derived vesicle called a phagosome, where they bombard bacteria pathogens with a complex mixture of reactive oxygen (ROS), nitrogen (RNS), and chlorine species [29]. This process is known as “oxidative burst,” and it is triggered by the NADPH oxidase complex (NOX2), which is critical for successful pathogen killing [31]. The phagosomal membrane helps in NOX2 assembling [31]. NOX2 is responsible for catalyzing the partial reduction of oxygen through the addition of one electron from NADPH to molecular oxygen, which results in the generation of superoxide anion (O_2_^•−^) [30]. The subsequent generation of H_2_O_2_ from O_2_^•−^ is catalyzed by the enzyme myeloperoxidase (MPO), which also helps convert H_2_O_2_ to hypochlorous acid (HOCL), a very powerful oxidant in vivo [29]. Bacteria, particularly the pathogenic ones, have developed excellent counterstrategies to survive the “oxidative burst” of the host [27]. Some bacteria control phagocytic cells to reduce their antibacterial activity. For example, neutrophils infected with *Francisella tularensis* do not generate ROS effectively because the bacteria hinder NOX2 assembly in the host [27,29].

The four major types of ROS in bacteria include O_2_^•−^, H_2_O_2,_^•^OH, and singlet oxygen (^1^O_2_) [32] (Table 1). These ROS have varying kinetics and degrees of activity [25,26], and due to the low reactivity and ability to be detoxified by natural antioxidants (enzymatic and nonenzymatic), O_2_^•−^ and H_2_O_2_ pose fewer risks than ^•^OH and ^1^O_2_ [32,33,34,35]. Because no enzyme can detoxify ^1^O_2_ and ^•^OH, these radicals are very poisonous and can be fatal [32,33,34]. Thus, oxygen plays a dual role in the aerobic metabolism of bacteria: it generates energy for bacteria, and its reduction via the electron transport system creates species (O_2_^•−^, ^•^OH, and H_2_O_2_) that are lethal to bacteria (Table 1). In host cells, together with unstable intermediates from lipid peroxidation, ROS have a significant impact on human health [32,33,34,35]. For instance, illnesses such as atherosclerosis, Alzheimer’s, and autoimmune disorders, among others, are caused by oxidative stress that occurs when the radical-scavenging systems of the body are overwhelmed [33,34,35].

**Table 1:** Stepwise reduction of ROS byproducts via electron transfer in bacterial cells.

ROS Species	Generation Steps and Characteristics of ROS	Stepwise Reduction Equation	References
hydroperoxyl radical (HO_2_^•^)	During a series of events during respiration, molecular oxygen is reduced to hydroperoxyl radical (HO_2_^•^).	O_2_ + **e**^−^ + H^+^ → HO_2_^•^	[15,25,36]
superoxide (O_2_^•−^)	The hydroperoxyl radical (HO_2_^•^) dissociates to form superoxide (O_2_^•−^). The reduction potential of this species is based on the environmental conditions of the solution. It can act as a mild oxidizing agent in an aqueous solution and, under other environmental conditions, it can act as a reducing agent.	HO_2•_ → H^+^ + O_2_^•−^ Fe^2+^ + O_2_ ↔ Fe(II)O2 ↔ Fe(III)O_2_^−^ ↔ Fe^3+^ + O_2_^•−^	[15,25,26,36]
Hydrogen peroxide (H_2_O_2_)	Superoxide (O_2_^•−^) undergoes additional transformations as a result of a well-known dismutation reaction. This process is hastened by the superoxide dismutase (SOD) enzyme, which has a copper-zinc core to generate hydrogen peroxide (H_2_O_2_). However, other enzymes (e.g., urate oxidase and glucose oxidase) have been shown to catalyze this reaction. It is critical that the cells “neutralize” H_2_O_2,_ since the species can be reduced through Fenton chemistry to ^•^OH, which is lethal.	O_2_^•−^ + 2H^+^ + **e**^−^ → H_2_O_2_ 2H+ + O_2_^•−^ + O_2_^•−^ → H_2_O_2_ + O_2_	[26,32,36,37]
Hydroxyl radical (^•^OH)	A hydroxyl radical (^•^OH) is generated through the Fenton reaction. Of note, in live organisms and at physiological pH, the ferrous ion (Fe^2+^) that accelerates the Fenton reaction has a short lifespan and can quickly auto-oxidize to ferric (Fe^3+^). The hydroxyl radical is a highly aggressive radical that can impede the correct functioning of a variety of biological molecules.	H_2_O_2_ + **e**^−^ → HO^−^ + ^•^OH H_2_O → ^•^OH + H^•^ + **e**^−^_aq_ → H_2_O_2_ Fe^2+^ + H_2_O_2_ → Fe^3+^ + ^−^OH + ^•^OH	[25,26,36]
Singlet oxygen (^1^O_2_)	Singlet oxygen is considered one of the most dangerous species of ROS generated through the natural process, with huge biological significance.	(a) Energy transfer to photosynthetic cells from light-sensitive molecule (b) Coexisting of oxygen and light in cells containing molecules such as flavins, quinones, porphyrins, etc. (c) Production through aromatic chemicals such as naphthalene, methylene blue, and several antimicrobial agents (d) Macrophage respiratory bursts (e) Lipid peroxidation activities	[33,36]

#### 2.1.2. Defense against ROS Generation in Bacteria

**Figure 2:**
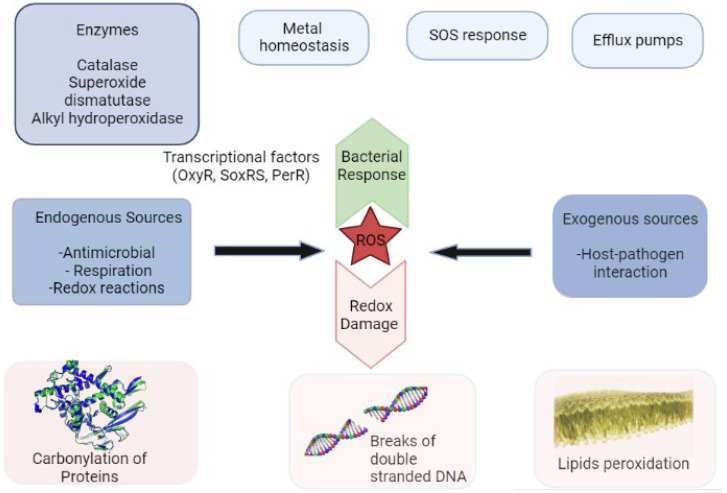
Antioxidant defense against ROS generation in bacteria that could originate from both exogenous and endogenous sources. Repair mechanisms as a means of bacterial response involving both enzymatic and non-enzymatic ways help ensure normal redox potential in bacteria, which are often controlled by transcriptional factors such as OxyR, PerR, and SoxRS. In the case of the repair mechanisms being overwhelmed, the oxidative stress (redox impact) is set such that it could damage bacteria macromolecules such as proteins, lipids, and deoxyribonucleic acid (DNA).

In *Escherichia coli*, the regulatory response to ROS (H_2_O_2_ and O_2_^•−^) generation is through the activation of superoxide dismutase (SOD) and catalase [15,38] (Figure 2). Unlike human cells with only two SOD isoforms, *E. coli* utilizes three distinct kinds of SOD with various metal cationic locations: CuZnSOD (sodC), FeSOD (sodB), and MnSOD (sodA) [10,38]. These enzymes catalyze the conversion of superoxide anion (O_2_^•−^) to hydrogen peroxide (H_2_O_2_), thereby limiting the accumulation of superoxide radicals in bacteria cells [10,15]. Catalases, on the other hand, catalyze H_2_O_2_ into H_2_O and O_2_, and *E. coli* possesses two types of catalases (hydroperoxidase I (HPI) and hydroperoxidase II (HPII)), which are also present in several other bacteria [39,40,41]. Alkyl hydroperoxidase in the bacterial cell has also been demonstrated to be important in limiting ROS generation by making use of two peroxidases (AhpD and AhpC) in its catalytic action [15,42]. The AhpC acts as the catalytic component, and AhpD works as an essential protein adaptor during metabolism [15]. Further, some small proteins that are referred to as thiols (e.g., glutathione, thioredoxin, and peroxiredoxin) in bacteria stimulate the breakdown of ROS, reactive nitrogen species (RNS), and their intermediates to repair oxidatively and nitrosatively damaged proteins [8,42]. Thiol’s lower cellular level of disulfide and glutathione, which is the most abundant, not only serves as a cofactor to glutathione peroxidase but also helps reversion of other non-enzymatic antioxidants, such as α-tocopherol and ascorbic acid, to their active form [8,42]. In addition to the general stress regulon (RpoS), SOD and catalase are controlled by OxyR and SoxRS regulons [43]. These have been studied extensively using *E. coli* as a model, and their impacts as SOD and catalase regulators on a wide range of bacterial phyla ranging from Actinobacteria to Proteobacteria have been reported [15,42]. In anaerobic bacteria that do not have catalase for H_2_O_2_ elimination, OxyR has been demonstrated to be important in their tolerance to H_2_O_2._ In addition, OxyR is involved in the defense against singlet oxygen and cellular damage caused by lipid peroxidation in bacteria [8,15,43]. Mutations in OxyR in *E. coli* have been shown to reduce cellular sensitivity to H_2_O_2_, resulting in an increase in one of the three enzymes (catalase hydroperoxidase I, catalase hydroperoxidase II, and alkyl hydroperoxide reductase) implicated in the elimination of organic ROS and H_2_O_2_ [8,16,44]. These three enzymes are controlled by the katG gene product, katEF, and Ahp genes, respectively [16,43]. SoxS, on the other hand, is critical to activating gene transcription to minimize superoxide and nitric oxide stress in bacterial cells, as well as regulating membrane permeability through efflux pump and outer membrane porin production [8,44]. Generally, defense against ROS in bacteria protects pathogenic bacteria against both the host immune response and antimicrobial therapy, which enable bacteria to cause chronic infections in the host.

#### 2.1.3. Mechanism of Bacterial Lethality via Oxidative Stress

**Figure 3:**
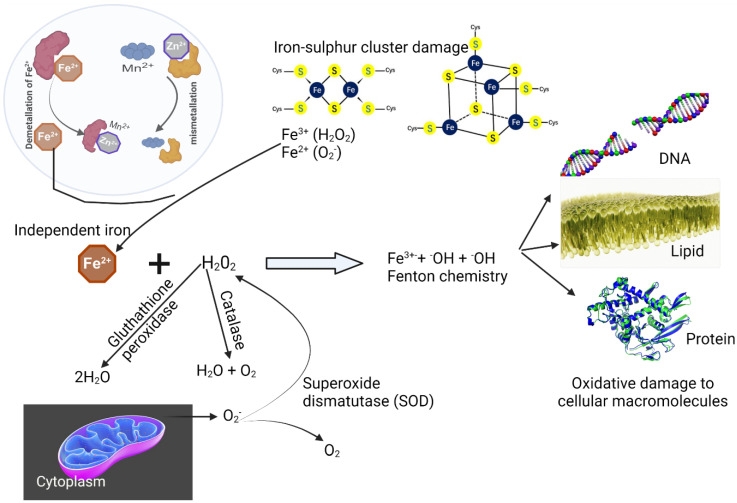
A multiscale explanation of the metabolism and a macromolecular example of how ROS causes macromolecule damage. ROS demetallize mononuclear Fe^2+^ proteins, which are then mismetallized with alternative divalent metal ions. Further, through damage to the iron-sulfur cluster, independent Fe^2+^ are generated. Independent Fe^2n+^ spontaneously interacts with H_2_O_2_ (produced via dismutase of superoxide (O_2_^−^)) via the Fenton reaction, releasing hydroxyl radicals that can damage bacterial macromolecules.

The term ROS refers to a group of highly reactive compounds that include molecular oxygen (O_2_) [8,25]. Electron reduction of oxygen during metabolism in the cell generates superoxide anion (O_2_^−^). Superoxide anion is a major ROS that can be further converted to H_2_O_2_ by SOD [15,43] (Figure 3). Hydroxyl radical (^•^OH) is produced through the Fenton reaction, which involves the reduction of H_2_O_2_. The Fenton reaction is normally activated through the disruption of iron homeostasis that results in an elevated amount of independent cellular Fe^2+^ [42] (Figure 3). Normal cells have a finely regulated and well-balanced redox state due to the continuous generation and detoxification of cellular ROS. However, exogenous stressors such as antibiotics can speed up the natural process of respiration or microbial metabolism of food, leading to a high generation of endogenous ROS that weakens the ROS defense mechanisms [8,16,44]. The subsequent degeneration of the defense systems due to overwhelming ROS production can lead to oxidative stress, which is lethal to cells, since ROS such as hydroxyl radicals’ accumulation can stimulate autoxidation of cellular macromolecules such as nucleic acids, lipids, and proteins, thereby altering their biological activity [10,11] (Figure 3). Autoxidation of cellular macromolecules (e.g., lipid peroxidation) is a process in which ROS remove electrons from macromolecules and subsequently generate reactive intermediates that can undergo further reactions. Bacterial cell membranes, or organelle membranes, are particularly susceptible to ROS damage, due to their high levels of polyunsaturated fatty acids. Lipid peroxidation damages bacterial phospholipids directly and can also act as a cell death signal-inducing programmed cell death. Thus, since no protein-based mechanism can detoxify the hydroxyl radicals, ROS induce their key antibacterial activity when stress becomes severe, leading to the self-destruction of bacteria cells [12,45,46].

Generally, oxidative stress can either be endogenous or exogenous [11]. Exogenous oxidative stress occurs as a result of host–pathogen interactions in bacteria, whereas antibiotics treatment, respiration, and intracellular redox reactions contribute to endogenous oxidative stress [10,11] (Figure 2). Enzymes such as catalase, SODs, alkyl hydroperoxide reductase, and glutathione peroxidase can detoxify ROS in bacteria and are controlled by regulons such as SoxRS, OxyRS, SOS, and PerR, which help counteract the damaging effect of ROS [8,16,44]. For instance, *E. coli* defends itself against oxidative stress by increasing the activity of SOD and catalase, which catalyze the dismutation of O_2_ and H_2_O_2_, respectively. Furthermore, the OxyR and SoxRS regulons in *E. coli* govern the oxidation response by transcriptionally regulating catalase and SOD in reaction to H_2_O_2_ and O_2_, respectively [44].

#### 2.1.4. Antibiotic-Mediated ROS Lethality

Antibiotics’ principal mechanisms of action in bacteria are an attack to cell wall (β-lactam antibiotics), protein synthesis (aminoglycosides), and DNA replication (fluoroquinolones) [47,48]. However, some investigations have found that antibiotics cause ROS generation by the overexcitation of electrons through the Krebs cycle and the release of iron, which activates Fenton chemistry [17,19,47,48]. Hence, antibiotics with dissimilar primary mechanisms of action were discovered to share a common secondary mechanism in the form of ROS generation [47,48]. Arriaga-Alba et al. [49] were the first to establish that oxidative stress generation by quinolones contributed to the antibiotic’s mechanism of action in *Salmonella typhimurium* killing. Thereafter, antibiotics such as aminoglycosides, β-lactams, rifampicin, and chloramphenicol have been demonstrated to induce ROS generation, resulting in bacteria lethality [17,50,51]. However, nitrofurantoin and polymyxin B are the two of the most commonly implicated antibiotics in ROS-mediated bacterial lethality [16]. Regardless of their unique targets, ROS-inducing antibiotics cause oxidative damage through the increase in bacteria respiration, which results in NADH depletion, iron-sulfur cluster instability, and iron misregulation [19]. For instance, using aminoglycoside antibiotics, Kohanski et al. [50] found that the formation of ^•^OH caused mistranslation and abnormal folding of membrane-associated proteins, resulting in a stress response. Further, topoisomerase inhibitors have their primary mechanisms of bacterial killing via DNA poisoning, lesion repair inhibition, and interfering with DNA replication [52]. Dwyer et al. [18] implicated the involvement of oxidative damage (mediated by O_2_^•−^ and ^•^OH) in the lethality of topoisomerase inhibitors. In another study by Foti et al. [53], bactericidal antibiotics caused cell death by oxidizing the guanine nucleotide pool. Dorsey-Oresto et al. [54] published the first proof of post-stress programmed cell death mediated by ROS in bacteria. They implicated the YihE protein kinase in *E. coli* as an essential protein responsible for the bacteria’s self-destructive response to fatal stress. YihE protein kinase is controlled by the Cpx envelope (a response system to stress), which, together with MazF toxin and superoxide, helps assist bacteria in deciding whether to live or die in response to stress. Although it has been proven that oxidative stress induced by some bactericidal antibiotics plays a vital role in bacterial cell death, a study by Kohanski et al. [50] has also shown that at sub-lethal levels, ROS play a key role in bactericidal antibiotic-induced mutagenesis. In addition, studies by Imlay [20], Keren et al. [21], and Liu and Imlay [22] have argued that the formation of ROS contributed to antibiotic lethality because antibiotics appear to act under anaerobic circumstances. Hence, studies associating ROS generation with antibiotics have been largely controversial, with various viewpoints undermining and solidifying the hypothesis presented in Table 2. Despite these contradictions, the metabolism in bacteria influences antibiotic potency (for example, metal homeostasis and iron-sulfur proteins) [28,47,55].

##### Two Commonly Implicated Antibiotics in ROS-Mediated Bacterial Lethality

**Nitrofurantoin**

**Figure 4:**
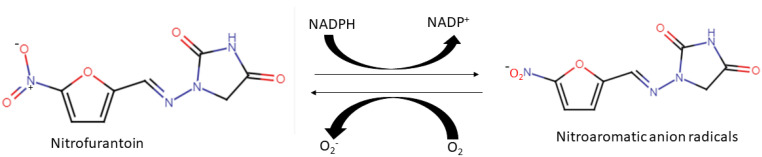
Redox cycling potential of nitrofurantoin, which demonstrates nitroaromatic anion formation through depletion of NADPH. In the presence of molecular oxygen, nitroaromatic anion generates superoxide anion.

Nitrofurantoin is a medication whose mechanism of action has been poorly understood since its discovery in the 1940s [56]. However, recent studies have implicated ROS generation as one of its major mechanisms of action [16]. The mechanism is based on cellular NADH depletion to create nitroaromatic anion radicals, which, in the presence of molecular oxygen (O_2_), often auto-oxidize to produce superoxide (O_2_^−^) [10,16] (Figure 4). The continued redox cycling of nitrofurantoin in bacteria cells results in the accumulation of O_2_^−^, which is often frequently converted to more lethal ROS (e.g., ^•^OH) that can damage bacteria cell macromolecules. The drug is employed in clinical practice to treat lower urinary tract infections (UTI) [10]. Because of nitrofurantoin’s quick infiltration into the lower urinary tract, the drug is only suitable for infections that affect the lower part of the urinary tract and would not work for other types of infections, as they would not achieve therapeutic concentrations [56]. This indicates that the basis for the adoption of the drug in clinical practice is seemingly based on its discriminating accessibility to the urinary tract [16]. The drug was approved by FDA in the 1950s and was widely used for the treatment of UTI infection until the 1970s, when other drugs such as β-lactams and trimethoprim-sulfamethoxazole became available [56]. However, recently, several procedures have declared nitrofurantoin to be the first-line treatment for UTI infections due to resistance to newer drugs [16]. Several researchers believe that the continued effectiveness and minimal resistance to the drug could be due to its minimal effects on bowel flora and its mechanism of action, which is based on ROS generation [56]. Hence, one advantage of ROS-inducing antimicrobial agents compared to other antibacterial polymeric materials could be their lower susceptibility to resistance.

**Polymyxin B**

Polymyxin B is a peptide-based antibacterial agent [57]. It is often employed in the treatment of infections caused by Gram-negative bacteria such as *Pseudomonas aeruginosa* [16]. However, as a result of some toxicity issues (neurotoxicity and nephrotoxicity) implicated in its usage, the drug is often reserved as a last resort for treatment [57,58]. The drug derives its potency via the accumulation of hydroxyl radicals, which consequently facilitate the disruption and loss of integrity of the cell membrane in Gram-negative bacteria. More crucially, PMB was found to be lethal to Gram-negative bacteria even at lower concentrations [58]. The ROS-mediated antibacterial action of PMB was brought to light through the revelation that PMB toxicity increases under aerobic conditions, as well as through deletion of the katA gene essential for H_2_O_2_ resistance in *P. aeruginosa* [57].

### 2.2. Natural and Synthetic Agents Contributing to ROS-Mediated Bacterial Lethality

Some natural agents such as phenolic compounds, honey, peptides, siderophores, and carotenoids, as well synthetic agents, have been linked to microbial mortality via ROS production. The mechanisms of action of these natural and synthetic antibacterial agents are presented in Table 3.

**Table 2:** Viewpoints undermining and solidifying the involvement of antibiotics in ROS-mediated bacterial lethality hypotheses.

Basis of Opinion	Views Undermining the Opinion	Studies Solidifying the Opinion
Involvement of ROS in antibiotics bacterial lethality	Iron/iron-sulfur clusters’ effect on bacterial killing by antibiotics is largely dependent on how antibiotics are taken up; ROS play no role [59]. Further, ROS accumulation and cell death in an antibiotic-treated cell are discordant [21]. Hydroxyl radical (^•^OH) accumulation did not often correspond to antimicrobial death [21].	Belenky et al. [60] demonstrated the involvement of ROS in antibiotic lethality by showing that cells exposed to antibiotics had cytotoxic changes including malondialdehyde adducts, protein carbonylation, double-strand DNA breaks, and nucleotide oxidation. which are indicative of ROS involvement. Further, the findings of Luan et al. [61] also laid justice to the involvement of ROS by demonstrating that katG mutants produce more ROS, which subsequently resulted in their high rate of death relative to the wild type after antibiotic treatment.
Type (antibiotics) and conditions that influence ROS generation	The ROS-mediated mechanism of killing is shared by all antibiotics, and anaerobiosis inhibits the lethality of norfloxacin (quinolone) at a low concentration. Furthermore, Iron chelator (dipyridyl) and hydroxyl radical scavenger (thiourea) prevented cells from being killed by antimicrobials under both aerobic and anaerobic conditions [21].	In 2014, Dwyer et al. [17] demonstrated that antibiotics belonging to fluoroquinolones, β-lactams, and aminoglycosides generate ROS while interacting with their target sites, though information on ROS generation of other classes of antibiotics is unconvincing. Further, findings from the study showed that ampicillin, gentamicin, and norfloxacin have only attenuated lethality under highly anaerobic conditions. Furthermore, Malik et al. [62,63] showed that the choice of norfloxacin by Keren et al. [21] was not a good candidate for ROS experiments due to the intermediate reactions to anaerobic cell death caused by quinolones.
Quantity of ROS generated	ROS produced during antibiotic treatment are too minute to truly kill bacterial cells [22].	The findings of Luan et al. [61] and Hong et al. [64], have, to a degree, debunked the claim on the quantity of ROS generation by demonstrating that stress can increase the number of sites available for ROS assault and that a high ROS concentration may not be required. Moreover, the findings of Luan et al. [61] and Dwyer et al. [17], through demonstration of stressors that created lesions that were hypersensitive to ROS attack, could also reduce the credibility of the argument. The actual evidence on the quantity of ROS generation relative to cell death came from Hong et al. [46] and Dorsey-Oresto et al. [54], who postulated that intracellular levels of ROS were capable of killing cells once the initial triggering stressor had been removed.
Detection of ROS	The low specificity of dyes for ROS detection and antibiotic therapy did not increase ROS [22].	The claim about low specificity of dyes for ROS was addressed by Dwyer et al. [17] in a ROS quantification experiment conducted in 2014, in which they used a wide range of fluorescent dyes to identify several kinds of ROS in bacteria, including H_2_O_2_, which could not be detected with the previously utilized HPF dye (3′-(p-hydroxyphenyl) fluorescein). In their findings, bactericidal agents including ampicillin, gentamicin, and norfloxacin increased H_2_O_2_ generation in bacteria after treatment.
Effectiveness of exogenous antioxidant	The possibility of chemical agents such as thiourea and dipyridyl causing off-target effects cannot be fully ruled out [20,22].	This causative argument about off-target effects of ROS scavenger could be countered by the findings of Luan et al. [61], who demonstrated that katG mutants produce more ROS, which subsequently resulted in their high rate of death relative to the wild type after antibiotic treatment.

#### 2.2.1. Phenolic Compounds

**Figure 5:**
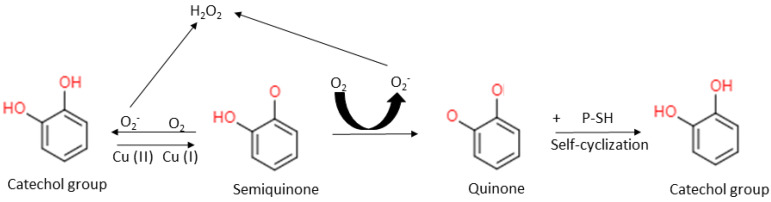
Redox cycling potential of catechol-bearing phenolic compounds, which demonstrates ROS and quinone formation in the presence of transition metal ions and oxygen and the subsequent reversion to catechol moieties through reaction with protein thiols (P-SH) or self-cyclization.

Because of their low redox potential, catechol-containing phenolic compounds could be excellent pro-oxidants that generate a considerable quantity of ROS through autoxidation in the presence of transition metals [45,65] (Figure 5). The ROS (O_2_^•−^ and H_2_O_2_) produced through this autoxidation are not highly reactive, and are hence less toxic to bacterial macromolecules [45,66]. However, the reaction of these ROS with independent ferrous ions in the cell via the Fenton reaction (Fe^2+^ + ^•^OH + ^•^OH) can produce a highly reactive hydroxyl radical (^•^OH) that causes damage to bacteria’s macromolecules and subsequent lethality [66] (Figure 3).

**Figure 6:**
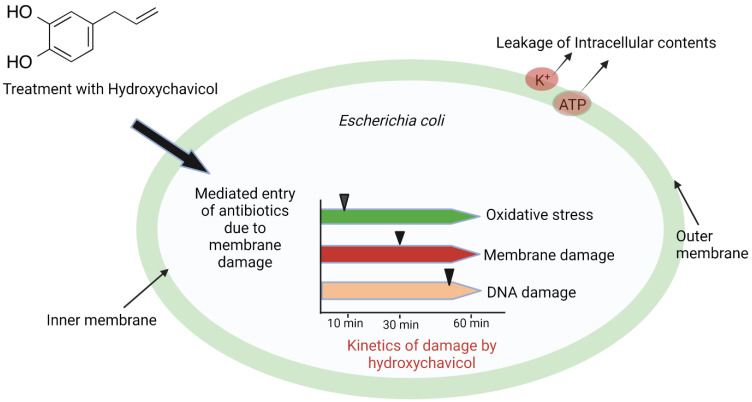
Antibacterial mechanisms of action of hydroxychavicol against *E. coli*.

Specifically, Singh et al. [67] established the capacity of hydroxychavicol isolated from *Piper betle* to kill *E. coli* via ROS induction and damage of DNA in *E. coli* cells. The study demonstrated that treatment of *E. coli* with hydroxychavicol in the presence of copper cleaves DNA and instigates DNA damage repair genes. Treatment in the presence of antioxidants protected cells against hydroxychavicol-induced ROS lethality, and damage to iron-sulfur proteins significantly amplifies oxidative stress instigated by hydroxychavicol. The *E. coli* wild type was less susceptible to hydroxychavicol treatment relative to the *E. coli gshA* mutant, which indicates the significant involvement of oxidative stress in the lethality of hydroxychavicol. Hydroxychavicol was found to be potent against other Gram-negative organisms isolated from persons with clinical symptoms and diverse resistance patterns. Later in 2021, Singh et al. [68], while working with the same compound, postulated the exact kinetics and series of events that occurred in the killing of *E. coli*. The study implicated the potential of hydroxychavicol to damage the *E. coli* cell membrane via oxidative stress as one of the earlier mechanisms involved in hydroxychavicol lethality. These findings showed that oxidative stress set in immediately after 10 min of treatment with hydroxychavicol and resulted in membrane damage at 30 min, followed by DNA breakage before 60 min (Figure 6). Genes involved in repairing DNA damage were not activated until after 60 min of treatment. Abnormal growth and cell breakage of *E. coli* were noted after 1 h of hydroxychavicol treatment at 125 μg/mL and 750 μg/mL, respectively. Cell porosity was observed to increase in the presence of magnesium ion (Mg^2+^), but this was halted in the presence of ethylenediaminetetraacetic acid (EDTA). The study concluded that hydroxychavicol pretreatment of Gram-negative organisms, which are most impervious to antibiotic treatment due to their outer membrane, could help in the treatment of infections caused by Gram-negative bacteria due to the damaging effects that hydroxychavicol had on the cell membrane.

Sinsinwar and Vadivel [69] worked on the antibacterial activity of catechin obtained from the shell nut of cashew against *Staphylococcus aureus* (both methicillin-sensitive and methicillin-resistant). The study revealed the minimum inhibitory concentration (MIC) of catechin against both methicillin-resistant and methicillin-sensitive *S. aureus* to be between 78.1 to 156.2 μg/mL. Following treatment with catechin, about 1.5-fold and 1.9-fold increases in ROS generation were noted in methicillin-sensitive and methicillin-resistant *S. aureus*, respectively, relative to the control (methicillin). Moreover, catechin treatment resulted in toxicity to the cell membrane and an increase in the leakage of nucleic acids (×18) and proteins (×16) relative to cells treated with methicillin. The specific activity of SOD and catalase drastically decreased from 9.63 to 5.31 U/mg and 3930 to 1573 U/mg, respectively, relative to the control. The study ascribed the effects of treatment with catechin to the involvement of oxidative stress in the killing of both methicillin-sensitive and methicillin-resistant *S. aureus*.

Ajiboye et al. [66] demonstrated the contribution of oxidative stress in the bactericidal activity of protocatechuic acid. Protocatechuic acid has an MBC value of 700, 800, and 800 μg/mL and a MIC value of 600 μg/mL each against *E. coli*, *P. aeruginosa*, and *S. aureus,* respectively. The protocatechuic acid-killing rate of bacterial cells was time-dependent, and the amount of ROS (superoxide anion) generated following treatment at 4× MIC increased significantly relative to the negative control (dimethyl sulfoxide (DMSO)-treated cells). Following treatment of bacterial cells with protocatechuic acid, SOD, NAD^+^/NADH, and catalase increased considerably relative to the negative control. Equally, there was a decrease in glutathione levels and an increase in glutathione disulfide, malondialdehyde, and damage of DNA following treatment of bacterial cells with protocatechuic acid relative to the negative control. All findings from the study indicate the generation of ROS (O_2_^•−^and ^•^OH) in the bactericidal activity of protocatechuic acid against *E. coli*, *P. aeruginosa*, and *S. aureus.* The study concludes that protocatechuic acid instigates ROS generation possibly through Fenton chemistry, autoxidation, and hampering of the electron transport chain, consequently resulting in the peroxidation of lipids, the breakage of DNA, and the ultimate cell death of the bacterial cell. In another study by Ajiboye et al. [70], the authors reported that phenolic acids (protocatechuic acid, gallic acid, and caffeic acid) increased colistin-mediated bacterial killing via the disruption of redox homeostasis. Making use of the wild and mutant strains, the antibacterial activity of all the phenolics showed that the wild type had higher MIC values relative to the sodB and katG mutant strains of *Acinetobacter* baumannii AB5075. Protocatechuic acid had the highest antibacterial activity among the phenolics, with a MIC of 32 g/mL against the sodB mutant strain and 64 g/mL against the katG mutant strain. The checkerboard analysis further showed that the phenolics could potentiate colistin antibacterial activity. Treatment with the phenolics revealed the generation of superoxide anion and an increase in the pair NAD+/NADH and ADP/ATP, which all pointed to the contribution of oxidative stress in the lethality to bacteria induced by the study phenolics. Similarly, a reduced level of glutathione was also noted, which further confirmed the involvement of oxidative stress in the killing of *A. baumannii.* The study concluded that via oxidative stress, the studied phenolic compounds enhanced the potency of colistin against *A. baumannii* AB5075.

Xiong et al. [71] studied the antibacterial properties of epigallocatechin gallate (EGCG) isolated from green tea against *E. coli*. Findings from the study demonstrated that the antibacterial activity of EGCG is not directly due to H_2_O_2_ generated by the compound but is rather a result of the increase in endogenous ROS and a reduced adaptive response to ROS in *E. coli*. It was also observed that EGCG acted synergistically with paraquat (a well-known pro-oxidant) in eliciting its effect. These observations substantiated the mechanism of antibacterial action of EGCG, which was previously unclear. However, the antibacterial effect of EGCG was impeded under anaerobic conditions, but it was concluded that EGCG increased intracellular oxidative stress in the inhibition of *E. coli* growth.

Hussain et al. [72] studied the effects of allyl pyrocatechol (AP), the main component in *Piper betle* ethanolic extract, on essential oxidative stress enzymes required for the survival of *S. aureus*, a significant pathogen in the human host. The nitroblue tetrazolium (NBT) reduction assay was used in the study to detect ROS by the chromogenic production of reduced formazan. This method makes it easier to distinguish between the quantities of ROS produced intracellularly and extracellularly. The absorbance values (A575 nm) for the NBT reduction test were 0.709 and 0.695 for untreated and AP-treated cells, respectively, at a concentration of 2 mg/mL MIC, indicating the existence of ROS. However, AP-treated *S. aureus* cells displayed decreased ROS levels both extracellularly and intracellularly relative to the untreated cells, but larger amounts of ROS stayed intracellularly at an absorbance of 0.457 relative to extracellular levels at an absorbance of 0.137 at *P* < 0.05. This observation is unlike previous studies in which catechol-containing phenolic compounds caused increased ROS relative to untreated cells [66,70]. However, this observation could have been due to the method employed in the study, as NBT is only reduced by O_2_^•−^, denoting that other ROS formed through the Fenton reaction were not considered [73]. This opinion was reinforced, as AP-treated *S. aureus* cells had higher *sodA* (1.5-fold) and *sodM* (0.7-fold) expression, with an equivalent increase in total SOD activity (12.24 U/mL) relative to the untreated cells (10.85 U/mL). Likewise, the transcription of ahpC was greatest in AP-treated cells with 5.5-fold upregulation compared to untreated cells at *P* < 0.05. Accordingly, ahpC activity was greater in AP-treated cells at 0.672 (A310 nm) compared to untreated cells, which were 0.394 (A310 nm). The findings revealed that AP’s pro-oxidant activity caused sufficient internal oxidative stress in *S. aureus*. As a result, *S. aureus* raised SOD expression and activity (*SodA, SodM*) to deal with the increased oxidative stress. The higher expression of *sodA* compared to *sodM* shows that the stress was induced internally by increasing O_2_^•−^.

#### 2.2.2. Honey

Surgihoney is a kind of honey for wound healing that has an antibacterial action that is based on high amounts of ROS in the form of H_2_O_2_. This honey is produced in the UK by Healing Honey International. Cooke et al. [74] investigated the antibacterial effects of Surgihoney and modified honey 1 and 2 (produced from *Apis mellifera*) against *S. aureus* and reported that the modified honeys had significantly higher antibacterial activity relative to Surgihoney. Surgihoney had a phenol activity of 31.5% relative to 63% and >63% observed in modified honey 1 and 2, respectively. Furthermore, higher H_2_O_2_ was generated by the modified honey relative to surgihoney over 24 h. The modified honey 1 and 2 produced 1 and 1.5 mM H_2_O_2_, respectively, versus 0.2 mM H_2_O_2_ produced by surgihoney over the same period. This observation demonstrates the correlation between the increase in phenol activity and the production of H_2_O_2_ which was correlated with the antibacterial activity noted in the study. The study concluded that the modified honey would afford a simple-to-apply, effective, and non-toxic wound-dressing agent.

#### 2.2.3. Carotenoid

Aribisala et al. [75] used in silico and in vitro approaches to examine the role of oxidative stress in astaxanthin-mediated bacterial mortality. It was observed that astaxanthin had a MIC of 8 μg/mL, which was lower than the 16 μg/mL observed for novobiocin against *S. aureus*. However, against *E. coli* and *P. aeruginosa*, novobiocin showed lower MICs of 0.25 and 0.125 μg/mL, respectively, compared to astaxanthin’s MIC of 16 g/mL against both species. Following further examination into the role of ROS in the antibacterial activity of astaxanthin, it was observed that treatment with astaxanthin stimulated increased superoxide anion production compared to DMSO-treated cells. The observed increase in ROS competed well with those elicited by the reference standards (novobiocin and ciprofloxacin) and was accompanied by both glutathione (GSH) depletion and a considerable increase in ADP/ATP ratio. Furthermore, a time-dependent inhibition of hydroxyl radicals in the presence of 2,2′dipyridyl drastically reduced the rate of killing of the tested organisms. These findings indicate the contribution of oxidative stress in astaxanthin bactericidal activity. The in vitro findings were also consistent with the in silico evaluations on topoisomerase 2As, a target implicated in facilitating ROS generation when interacting with fluoroquinolones. The study concluded that astaxanthin could be developed as a novel natural topo2A inhibitor that has antibacterial activity based on ROS production.

#### 2.2.4. Antimicrobial Peptide

Using the *Mo*-CBP_3_ (MC) amino acid sequence isolated from the seeds of *Moringa oleifera* as the starting peptide, Oliveira et al. [76] designed three peptides (MC-PepI, MC-PepII, and MC-PepIII) and evaluated their antibacterial activities against two Gram-positive (*Bacillus subtilis* and *S. aureus*) and two Gram-negative bacteria (*E. coli* and *Klebsiella pneumoniae*). Findings from the study showed PepIII to be the most potent among the three peptides, and it had the highest potency against *S. aureus,* with an MIC_50_ value of 4.4 μM. Using *C. parapsilosis* as a model, it was demonstrated that the mechanism of action of PepIII (the most active peptide) functioned via an elevated accumulation of ROS and the incorporation of propidium iodide following treatment. The scanning electron microscopy analysis demonstrated damage to the cell membrane, consequently leading to cytoplasm content leakage and cell death. Furthermore, the three peptides demonstrated against human red blood cells did not cause hemolysis, and PepIII (the most potent peptide), when demonstrated against Vero cells even at a higher concentration (57 × MIC_50_ of S. *aureus*), did not result in cytotoxicity. The study concluded that the observations, however, demonstrated PepIII as a prospective antimicrobial candidate for bacterial and fungal infections.

#### 2.2.5. Siderophore

**Figure 7:**
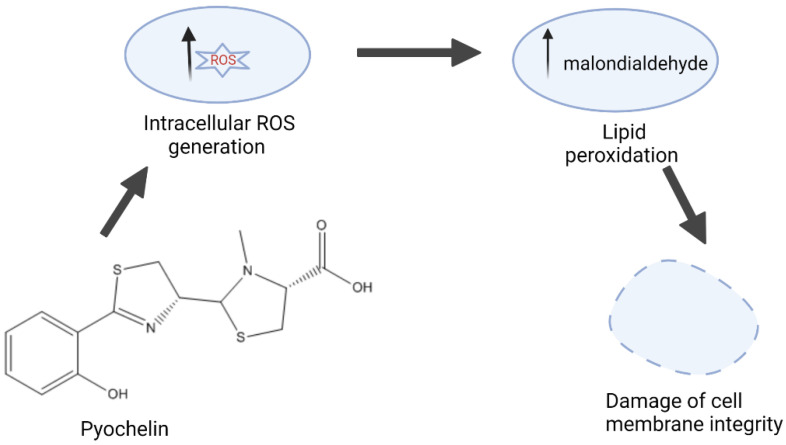
Pyochelin antibacterial mechanism of action.

Ong et al. [77] worked on the antibacterial activity of pyochelin, a siderophore isolated from *Burkholderia paludis*. The antibacterial activity was carried out against *S. aureus* and *E. faecalis*. Findings from the study showed *Enterococcus faecalis* (MIC of 3.13 µg/mL) to be more susceptible relative to *S. aureus* (MIC of 6.26 µg/mL). Following 24 h treatment with 1, 2, and 4 × MIC of pyochelin, an elevated increase in ROS in *E*. *faecalis* was noted and caused an increase in malondialdehyde levels, suggesting lipid peroxidation relative to the control (Figure 7). Furthermore, cell membrane disruption and cytoplasmic content leakage resulted in the cell death of the organism. When *E. faecalis* was treated for 24 h with 2 and 4 × MIC of the siderophore, the survival rate of the organisms was drastically reduced by roughly 80% relative to the negative control. The study concluded that pyochelin could be developed as therapeutics for infections caused by *E. faecalis* and *S. aureus* due to pyochelin’s capability to generate endogenous ROS in bacterial cells.

#### 2.2.6. Synthetic Agents in ROS-Mediated Bacterial Lethality

Wang et al. [78] worked on the photobiological activities of six derivatized cationic complexes ((i), phenanthroline, (ii), 3,8dipyrenylphenanthroline, (iii) 3,8dipyrenylphenanthroline, (iv) 3-phenylphenanthroline, (v) 3-pyrenylphenanthroline, and (vi) 3,8diphenylethynylphenanthroline) containing heteroleptic (III) with tris-diimine moiety in vitro. Findings from their study revealed that complexes 3 and 5 at 81 and 72%, respectively, had the highest quantum yield of singlet oxygen among all studied derivatives. The derivatives had long-lived triplet excited states, which allowed for intramolecular contact with ground-state oxygen and significant ROS generation. Following photoactivation and after treatment with derivatives 3 and 5, elevated production of ROS in SK-MEL-28 (human malignant cells) was observed relative to the dark control and tert-butyl hydrogen peroxide (positive control). Furthermore, after photoactivation, derivatized 3 and 5 demonstrated significantly enhanced antibacterial activity against the studied strains *S. aureus* (EC_50_ of 0.17 µM) and *S. mutans* (0.18 µM) relative to the dark condition, with a mean EC_50_ of 11.2 µM and 5.59 µM for *S. mutans* and *S. aureus* respectively. They concluded that the derivatives could be promising photoactivation agents against pathogenic bacteria through their capability of eliciting ROS.

Wang et al. [79] developed a light-responsive T-TCP micelle and determined its antibacterial activity against bacterial biofilms. The T-TCP micelle is a self-assembling amphiphilic copolymer comprising thymol, toluidine blue O (TBO), grafted chitosan, and propylene sulfide (PPS). When the T-TCP micelle was irradiated with a 670 nm light source, about a four-fold higher fluorescence intensity of singlet oxygen sensor green was observed relative to the non-irradiated T-TCP, whose fluorescence intensity of singlet oxygen sensor green remain the same. A dramatic increase from 5 to 57% in transmittance was observed with the T-TCP micelle that was irradiated. The ROS generation under irradiation of T-TCP micelles by light activation of TBO was evidenced by the oxidation of thioether (hydrophobic) to sulfoxide (hydrophilic). Treatment of *S. aureus* and *Listeria monocytogenes* biofilms with irradiated T-TCP significantly reduced the formation of biofilms and killed almost all bacterial cells relative to the non-irradiated T-TCP, and it was observed that the reduction in biofilm formation and killing of cells was due to the drug release and ROS generated due to light activation of TBO. Cell wall damage was observed in cells treated with both T-TCP and T-TCP with thymol relative to the control (thymol), which did not result in cell wall damage of *S. aureus* after treatment. They concluded that the developed micelles could be employed as an antibacterial agent for disinfection.

Song et al. [80] worked on the antibacterial activity of the synthesized bis-quaternary ammonium salt (SBQA), a high molecular weight, long-chain organic compound. The antibacterial activity was carried out on two pathogenic bacteria (*S. aureus* and *E. coli*), and findings from their study showed that *S. aureus* (MIC value range 8–32 μg/mL) was more susceptible to SBQA than *E. coli* (16–64 μg/mL). Further, the rate of cell death was concentration-dependent, with a death rate increase from 40% to 99% as the concentration increased from 12.5 μg/mL to 200 μg/mL. At the same concentration of 25 μg/mL, SBQA had higher inhibitory effects on both *S. aureus* (95%) and *E. coli* (74%) than the positive control (CTAB) (55% and 50% killing rate for *E. coli* and *S. aureus,* respectively). SEM and TEM examination of bacterial cells treated with 200 μg/mL of SBQA revealed damage to the bacterial cell wall and a subsequent leakage of protein. The observed leakage of cytoplasm content was due to the ability of SBQA to induce ROS production, which causes damage to the cell membrane. The increase in ROS generation correlates with an increase in the concentration of SBQA administered, as revealed by the increased intensity of green fluorescence. Relative to the control group, a greater-than five- and four-fold increase in ROS generation was observed against *S. aureus* and *E. coli,* respectively, with no signs of cytotoxicity on the growth of mouse breast cancer cells (4T1) at a concentration lesser than 50 μg/mL. The authors concluded that SBQA could serve as a promising therapeutic drug candidate for bacterial infections.

**Figure 8:**
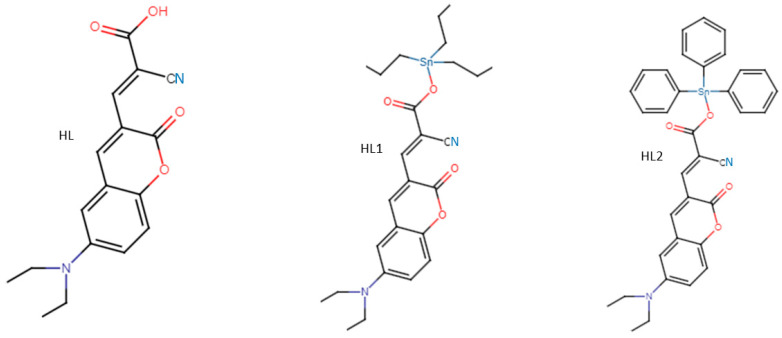
2D structure of HL, HL1, and HL2.

Hu et al. [81] derivatized two derivatives from HL, HL1 and HL2 (Figure 8) and examined their antibacterial potency against *E. coli* and *B. subtilis.* Their findings showed that the two derivatives had potent antibacterial activity relative to the parent compound HL due to the attached Sn atom (Figure 8). Among the derivatives, HL1 had the highest antibacterial activity against both organisms, and it was observed that HL1 alky chain flexibility and the compound lipophilicity enable its easy absorption through the cell membrane and, in so doing, cause damage. Relative to kanamycin (MIC 8 μg/mL), which serves as the positive control, HL1 showed a higher antibacterial effect with a MIC of 2 μg/mL. Against HELF, a human lung fibroblast, HL1 had a negligible cytotoxic effect even when the concentration was raised to 10 μg/mL and evaluated over a 24 h period. SEM evaluation of the treated cells (2 h) revealed that HL1 was able to pass through the *E. coli* cell membrane and enter the cell, causing cell membrane damage and leakage of cytoplasm content. In *B. subtilis* and *E. coli* treated with HL1, a significant amount of fluorescence was observed as a result of ROS generation, which could lead to oxidative stress and cause damage to the cell membrane and ultimately cell death. The authors concluded that ROS generation and subsequent damage caused by the release of ROS could be the key mechanisms employed by HL1 in bacterial killing and that the compound could be further developed and used for the treatment of infections caused by bacteria.

Liu et al. [82] designed a catechol-modified chitosan film (similar to melanin capsules produced by the immune system of insects in response to infection) and determined its in vitro antibacterial activity against *S. aureus* and *E. coli* and its in vivo wound-healing efficiency. Findings revealed that the catechol-chitosan film could accelerate electron transfer from redundant ascorbate to molecular oxygen to ensure continuous ROS production. After treatment with 1 and 10 mM of ascorbate, non-modified chitosan, oxidized catechol-chitosan film, and the reduced catechol-chitosan film had no sign of cytotoxicity on human keratinocyte cells. However, when the concentration of ascorbate used for treatment was increased to 300 mM, a 15% reduction in cell viability was observed. In the in vivo assay (exploring the subcutaneous administration model in rats), relative to the non-modified chitosan, a two-log average reduction in *S. aureus* viable count was noted in rats treated with catechol-chitosan film (reduced with 300 mM ascorbate) after three days. Moreover, the wounds of rats treated with catechol-chitosan film reduced with 10 mM ascorbate showed remarkable closure, with more evenly distributed collagen and the development of more epithelium and blood vessels after 14 days relative to wounds treated with chitosan alone or with oxidized catechol-chitosan film, which shows increased inflammation. A higher reduction in the bacterial count was also noted in rats treated with reduced catechol-chitosan film relative to other studied groups, correlating with the in vitro assay findings. The authors concluded that the reduced catechol-chitosan film had the potential for wound healing through ROS generation, which prevents bacteria invasion.

**Table 3:** Recent studies on natural and synthetic compounds supporting ROS-mediated bacterial killing.

Agent Commpound Classes	Agents	Bacteria	Implicated ROS and Mechanisms of Involvement in Antibacterial Activity	References
	**Natural agents**			
**Phenolics**	(1) hydroxychavicol	*E. coli*	Time-dependent destruction of cellular macromolecules by oxidative stress caused by hydroxyl radical (^•^OH). Destruction of the cell membrane occurs 30 min after treatment, and DNA damage onset occurs after 30 min.	[67,68]
	(2) Catechin	*S. aureus* (both methicillin-sensitive and methicillin-resistant)	Increased generation of ROS (O_2_^•−^ and H_2_O_2_) resulted in the onset of oxidative stress and subsequent damage to the cell membrane, nucleic acid, and protein by hydroxyl radical (^•^OH).	[69]
	(3) Epigallocatechin gallate	*E. coli*	Increased endogenous hydrogen peroxide (H_2_O_2_), leading to inhibition of *E. coli* growth	[71]
	(4) protocatechuic acid, gallic acid, and caffeic acid	*A. baumannii* (wild, sodB, and katG mutants)	Elevated superoxide anion (O_2_^•−^) production by the phenolic acids resulted in increased colistin-mediated bacterial killing via the destruction of redox homeostasis.	[70]
	(5) protocatechuic acid	*E. coli*, *P. aeruginosa*, and *S. aureus*	Protocatechuic acid instigates ROS (O_2_^•−^, H_2_O_2,_^•^OH) generation through Fenton chemistry, autoxidation, and hampering of electron transport chain, consequently resulting in peroxidation of lipid, breakage of DNA, and ultimate cell death of bacterial cells.	[66]
	(6) Allyl pyrocatechol	*S. aureus*	Allyl pyrocatechol provoked internal oxidative stress in *S. aureus* (O_2_^•−^), thereby amplifying the transcription and activities of SODs (*SodA, SodM*) in *S. aureus* to adapt to the increased oxidative stress.	[72]
**Honey**	(1) Surgihoney	*S. aureus*	Hydrogen peroxide (H_2_O_2_)	[74]
	(2) Modified honey 1	*S. aureus*	The elevated quantity of hydrogen peroxide (H_2_O_2_) resulted in higher antibacterial activity.	
	(3) Modified honey 2	*S. aureus*	The elevated quantity of hydrogen peroxide (H_2_O_2_) resulted in higher antibacterial activity.	
**Carotenoid**	Astaxanthin	*S. aureus* *E. coli* *P. aeruginosa*	ROS accumulation of superoxide anion (O_2_^•−^) and hydroxyl radical (^•^OH) causes a reduction in cellular glutathione level and increases the rate of bacterial death.	[75]
**Antimicrobial peptide**	(1) Mo-CBP3-PepI, II and III	Bacteria (*K. pneumoniae*, *B. subtilis, S. aureus, and E. coli*) and fungi (*C. tropicalis and C. albicans*)	Elevated accumulation of ROS (H_2_O_2_, O_2_^•−^, ^•^OH) causes loss of cell membrane integrity and ultimate cell death.	[76]
**Siderophore**	pyochelin	*E. faecalis* *S. aureus*	Elevated ROS production (O_2_^•−^, H_2_O_2_, ^•^OH) causes lipid peroxidation and cell death.	[77]
	**Synthetic agents**			
**Organic cationic salts**	Bis-quaternary ammonium salt	*S. aureus* *E. coli*	Induced ROS (O_2_^•−^, H_2_O_2_, ^•^OH) cause cytoplasm content leakage and cell membrane damage.	[80]
**Synthetic organic cationic complexes**	Derivatives of cationic heteroleptic (III) complexes with tris-diimine ligands moiety. (i) phenanthroline (ii) 3,8-diphenylphenanthroline (iii) 3,8dipyrenylphenanthroline (iv) 3-phenylphenanthroline (v) 3-pyrenylphenanthroline (vi) 3,8diphenylethynylphenanthroline	*S. aureus* *S. mutans*	Photoactivation of singlet oxygen (^1^O_2_) against pathogenic bacterial cells	[78]
**Amphiphilic copolymer**	T-TCP micelles (comprising of propylene sulfide and toluidine blue O (TBO) grafted chitosan)	*S. aureus* *L. monocytogenes*	Potent antibacterial activity due to ROS (H_2_O_2_, O_2_^•−^, ^•^OH) produced by TBO activation	[79]
**Organotin complexes**	Organotin complexes (1) HL1 (2) HL2	*E. coli* *B. subtilis*	An increase in the amount of accumulated ROS (H_2_O_2_, O_2_^•−^, ^•^OH) caused membrane damage and leakage of cytoplasm content in the organism.	[81]
**Modified chitosan**	Catechol-modified chitosan with melanin capsule	Methicillin-resistant *S. aureus* (MRSA) *S. aureus* *E. coli*	Electron transfer catalyzed by Catechol-modified film from ascorbate to molecular oxygen instigated continued ROS generation (H_2_O_2_, O_2_^•^) and triggered improved antibacterial activities in vitro and in vivo.	[82]

### 2.3. Safety Concerns Associated with ROS as a Contributory Antimicrobial Agent

The concept of ROS-mediated microbial killing has long been exploited in disinfectants and antiseptics but has only recently been identified in therapeutic applications, hence the safety and toxicity concern related to its usage is of great relevance. Studies on ROS-induced antibacterial agents have demonstrated an increase in potency at higher concentrations of ROS [39,74,83]. However, the potential risk of toxicity to host cells and tissues at such a high concentration is anticipated and hence should be prudently considered. ROS have been linked to mutagenesis. A study by Kohanski et al. [50] directly linked ROS with mutagenesis at sub-lethal levels, and through the blockage of ROS generation, mutagenesis was avoided. Furthermore, no protein-based mechanism can detoxify the hydroxyl radical (^•^OH), which can cause oxidative damage to several cells and tissues of the body made up of lipids, proteins, DNA, and carbohydrates [8,84]. The skin, which is made up of polyunsaturated fatty acids, is one of the most susceptible targets of ROS, where it can initiate lipid peroxidation [15]. For instance, cellular ROS generation has been shown to correlate with cytotoxicity in HFF-1 (human skin cells) [82] and HaCaT (human keratinocyte cells) [42,85]. Further, following treatment with particulate matter, findings show stress to the endoplasmic reticulum, mitochondrial damage, autophagy, and apoptosis in human keratinocytes, and mice with hairless skin tissues were reported to be due to ROS production [86]. Furthermore, in human keratinocytes and mouse skin, Jin et al. [87] noticed that particulate matter produced inflammation via ROS-mediated activation of inflammatory cytokines IL-8 and matrix metalloproteinase1 (MMP-1). However, using ROS with a lower redox potential (such as H_2_O_2_), as previously used in Surgihoney by Healing Honey International, could be the way to go for the treatment of infections topically [74]. Relative to hydroxyl radicals (^•^OH), hydrogen peroxide is less reactive and can be more easily scavenged by competent cells in the body.

Interestingly, studies showing non-toxicity issues due to treatment with ROS on human tissues and cells have also been reported over the years. Hu et al. [81] reported on two derivatives (HL1 and HL2) from HL (Figure 8) and demonstrated the ability of HL1 (the most effective among the derivatives) against HELF (human lung fibroblast). Findings from the study showed negligible cytotoxic effects even when the concentration was raised to 10 μg/mL (5 × MIC) over a 24 h period. Similarly, Song et al. [80] worked on the antibacterial activity of SBQA (synthesized bis-quaternary ammonium), a high-molecular weight, long-chain organic compound, and noticed no signs of cytotoxicity on the growth of mouse breast cancer cells (4T1) at concentrations lesser than 50 μg/mL. Using the *Mo*-CBP_3_ (MC) amino acid sequence isolated from the seeds of *Moringa oleifera* as the starting peptide, Oliveira et al. [76] designed three peptides (*M*C-PepI, *M*C-PepII, and *M*C-PepIII) and evaluated their toxicity against human red blood cells. Findings from the study showed no sign of hemolysis, and when PepIII (the most potent peptide) was demonstrated against Vero cells even at a higher concentration of 57 × MIC of *S. aureus* and 64 × MIC of *Candida* spp., no sign of cytotoxicity issues was observed.

Hence, in the use of ROS as an effective therapeutic strategy in humans for the treatment of microbial infections, considerable effort must be put into the appropriate quantification of ROS generated by antimicrobials and the determination of the minimum concentration that can elicit toxicity in host cells and tissues. However, the determination of such concentrations might be problematic, as the ROS-scavenging capability of patients differs and is controlled by factors relating to age, pregnancy, immune system, and underlying ailments such as diabetes.

## 3. Conclusions and Perspectives

The use of ROS-inducing antimicrobial agents to treat pathogenic infections is a promising and emerging field that provides an alternative viable strategy for antibacterial medication development and enhancement. This approach will not only broaden the scope of the antibiotic arsenal but also help fight against the menace of antibiotic resistance that has become a major public health concern. For the successful use of this approach, some of the limitations, such as toxicity issues associated with ROS (most especially the hydroxyl radical) and the quantification of ROS for the appropriate determination of ROS generated by each agent, need to be swiftly addressed. ROS cannot discriminate between bacterial and mammalian cells and, like microorganisms, most human cells have limited defense against hydroxyl radicals. Hence, the mechanism through which ROS contribute to antimicrobial lethality, which is based on hydroxyl radical generation during oxidative stress that often causes damage to critical bacterial macromolecules (lipids, proteins, and DNA), and subsequent death may also cause damage to the host cell. In humans, oxidative stress is treated with antioxidants such as vitamin E, ascorbic acid, selenium, carotenoids, and lycopene, among others. However, clinical trials on the scavenging capability of hydroxyl radicals with such antioxidants have failed to produce the desired results over the years [88]. Hence, future research on more potent antioxidants against hydroxyl radicals will be a boost to the full implementation and usage of novel antibacterials employing the involvement of ROS against human pathogenic infections. However, recent findings on nanoenzymes, specifically silver-palladium bimetallic alloys, showed their ability to generate surface-bound ROS that selectively kills bacterial cells over mammalian cells [89]. Interestingly, the nanoenzyme was potent against antibiotic-resistant bacteria while also delaying the development of antibiotic resistance in bacteria. This, however, suggests another viable approach for the utilization of ROS for the treatment of infections.

The issue of resistance in bacteria due to the ability of ROS to cause mutagenesis is another limitation. However, since combination therapy of ROS-inducing novel antibacterials with conventional antibiotics (such as isoquercitrin in combination with AmB and FLC [90] and kanamycin in combination with alanine [91]) has been shown to show promise to achieve enhanced antibacterial efficacy, such resistance in bacteria could be managed. Similarly, more research on the capability of ROS-inducing agents to act simultaneously on more than one target in bacteria could also help in the management of resistance. Overall, ROS-inducing antimicrobial agents have demonstrated increased potency at higher ROS concentrations, but the potential toxicity risk to host cells and tissues is anticipated. Hence, the prudent use of this approach for treatment should be carefully considered to reduce cytotoxicity issues and other adverse effects in patients. In doing this and to reduce resistance in bacteria, combination therapy of conventional antibiotics and ROS-inducing agents could be employed. Furthermore, the use of less reactive ROS such as H_2_O_2_ (as previously employed in Sugihoney) could also be employed for the treatment of infections topically, as H_2_O_2_ can be more easily scavenged by competent cells in the body.

## Data Availability

The data are contained within the article.

## References

[1] Larsson G.D.G.J., Flach C.F. (2022). Antibiotic resistance in the environment. Nat. Rev. Microbiol..

[2] Kohler V., Vaishampayan A., Grohmann E., Ahmad I., Ahmad S., Rumbaugh K. (2019). Problematic Groups of Multidrug-Resistant Bacteria and Their Resistance Mechanisms. Antibacterial Drug Discovery to Combat.

[3] Malik B., Bhattacharyya S. (2019). Antibiotic drug-resistance as a complex system driven by socio-economic growth and antibiotic misuse. Sci. Rep..

[4] Nelson R.E., Slayton R.B., Stevens V.W., Jones M.M., Khader K., Rubin M.A., Jernigan J.A., Samore M.H. (2017). Attributable mortality of healthcare-associated infections due to multidrug-resistant gram-negative bacteria and methicillin-resistant Staphylococcus aureus. Infect. Control Hosp. Epidemiol..

[5] World Health Organization Stop Using Antibiotics in Healthy Animals to Prevent 666 the Spread of Antibiotic Resistance. https://www.who.int/news-room/detail/07-11-2017-stop-using-antibiotics-in-healthy-animals-to-prevent-the-spread-of-antibiotic-resistance.

[6] Rather I.A., Kim B.C., Bajpai V.K., Park Y.H. (2017). Self-medication and antibiotic resistance: Crisis, current challenges, and prevention. Saudi J. Biol. Sci..

[7] Ventola C.L. (2015). The antibiotic resistance crisis: Part 1: Causes and threats. Phys. Ther..

[8] Lam P.L., Wong R.S.M., Lam K.H., Hung L.K., Wong M., Yung L.H., Ho Y.W., Wong W.Y., Hau D.K.P., Gambari R. (2020). The Role of Reactive Oxygen Species in the Biological 1 Activity of Antimicrobial Agents: An Updated Mini Review. Chem.-Biol. Interact..

[9] Mourenza A., Gil J.A., Mateos L.M., Letek M. (2020). Oxidative stress-generating antimicrobials, a novel strategy to overcome antibacterial resistance. Antioxidants.

[10] Kim S.Y., Park C., Jang H.J., Kim B.O., Bae H.W., Chung I.Y., Kim E.S., Cho Y.H. (2019). Antibacterial strategies inspired by the oxidative stress and response networks. J. Microbiol..

[11] Dryden M. (2018). Reactive oxygen species: A novel antimicrobial. Int. J. Antimicrob. Agents.

[12] Wang X., Zhao X. (2009). Contribution of oxidative damage to antimicrobial lethality. Antimicrob. Agents Chemother..

[13] Alvarado A., Arce I. (2016). Antioxidants in respiratory diseases: Basic science research and therapeutic alternatives. Clin. Res. Trials.

[14] Fanjul-Moles M.L., López-Riquelme M.L. (2016). Relationship between oxidative stress, circadian rhythms, and AMD. Oxidative Med. Cell. Longev..

[15] Vatansever F., De Melo W.C., Avci P., Vecchio D., Sadasivam M., Gupta A., Chandran R., Karimi M., Parizotto N.A., Yin R. (2013). Antimicrobial strategies centered around reactive oxygen species—Bactericidal antibiotics, photodynamic therapy, and beyond. FEMS Microbiol. Rev..

[16] Vaishampayan A., Grohmann E. (2022). Antimicrobials Functioning through ROS-Mediated Mechanisms: Current Insights. Microrganisms.

[17] Dwyer D.J., Belenky P.A., Yang J.H., MacDonald I.C., Martell J.D., Takahashi N., Chan C.T.Y., Michael A.L., Braff D., Schwarz E.G. (2014). Antibiotics induce redox-related physiological alterations as part of their lethality. Proc. Natl. Acad. Sci. USA.

[18] Dwyer D.J., Kohanski M.A., Hayete B., Collins J.J. (2007). Gyrase inhibitors induce an oxidative damage cellular death pathway in *Escherichia coli*. Mol. Syst. Biol..

[19] Kohanski M.A., Dwyer D.J., Hayete B., Lawrence C.A., Collins J.J. (2007). A common mechanism of cellular death induced by bactericidal antibiotics. Cell.

[20] Imlay J.A. (2015). Diagnosing oxidative stress in bacteria: Not as easy as you might think. Curr. Opin. Microbiol..

[21] Keren I., Wu Y., Inocencio J., Mulchay L.R., Lewis K. (2014). Killing by bactericidal antibiotics does not depend on reactive oxygen species. Science.

[22] Liu Y., Imlay J.A. (2013). Cell death from antibiotics without the involvement of reactive oxygen species. Science.

[23] Moher D., Liberati A., Tetzlaff J., Altman D.G. (2009). Preferred reporting items for systematic reviews and meta-analyses: The PRISMA statement. PLoS Med..

[24] Lobritz M.A., Belenky P., Porter C.B., Gutierrez A., Yang J.H., Schwarz E.G., Dwyer D.J., Khalil A.S., Collins J.J. (2017). Antibiotic efficacy is linked to bacterial cellular respiration. Proc. Natl. Acad. Sci. USA.

[25] Finnegan M., Linley E., Denyer S.P., McDonnell G., Simons C., Maillard J.Y. (2010). Mode of action of hydrogen peroxide and other oxidizing agents: Differences between liquid and gas forms. J. Antimicrob. Chemother..

[26] Forman H.J., Torres M. (2001). Signaling by the respiratory burst in macrophages. IUBMB Life.

[27] Van Acker H., Coenye T. (2017). The role of reactive oxygen species in the antibiotic-mediated killing of bacteria. Trends Microbiol..

[28] Stokes J.M., Lopatkin A.J., Lobritz M.A., Collins J.J. (2019). Bacterial Metabolism and Antibiotic Efficacy. Cell Metab..

[29] Winterbourn C.C., Kettle A.J., Hampton M.B. (2016). Reactive oxygen species and neutrophil function. Annu. Rev. Biochem..

[30] Mortaz E., Alipoor S.D., Adcock I.M., Mumby S., Koenderman L. (2018). Update on neutrophil function in severe inflammation. Front. Immunol..

[31] Müller A., Langklotz S., Lupilova N., Kuhlmann K., Bandow J.E., Leichert L.I.O. (2014). Activation of RidA chaperone function by N-chlorination. Nat. Commun..

[32] Chen P.R., Brugarolas P., He C. (2011). Redox signaling in human pathogens. Antioxid. Redox Signal..

[33] Glaeser J., Nuss A.M., Berghoff B.A., Klug G. (2011). Singlet oxygen stress in microorganisms. Adv. Microb. Physiol..

[34] Altman N. (2007). The Oxygen Prescription: The Miracle of Oxidative Therapies.

[35] Droge W. (2002). Free radicals in the physiological control of cell function. Physiol. Rev..

[36] Buettner G.R. (1993). The pecking order of free radicals and antioxidants: Lipid peroxidation, alpha-tocopherol, and ascorbate. Arch. Biochem. Biophys..

[37] Authen R.L., Davis J.M. (2009). Oxygen toxicity and reactive oxygen species: The devil is in the Details. Pediatr. Res..

[38] Kim S.Y., Kim E.J., Park J.W. (2002). Control of singlet oxygen-induced oxidative damage in *Escherichia coli*. J. Biochem. Mol. Biol..

[39] Drlica K., Zhao X. (2021). Bacterial death from treatment with fluoroquinolones and other lethal stressors. Expert Rev. Anti-Infect. Ther..

[40] Memar M.Y., Ghotaslou R., Samiei M., Adibkia K. (2018). Antimicrobial use of reactive oxygen therapy: Current insights. Infect. Drug Resist..

[41] Szczepaniak P., Laskowska E. (2010). Antibiotics promoting oxidative stress inhibit formation of Escherichia coli biofilm via indole signaling. Res. Microbiol..

[42] Li H., Zhao X., Huang Y., Liao B., Cheng L., Ren B. (2021). Reactive Oxygen Species in Pathogen Clearance: The Killing Mechanisms, the Adaption Response, and the Side Effects. Front. Microbiol..

[43] Akhova A.V., Tkachenko A.G. (2020). Role of Secondary Oxidative Stress in the Bactericidal Action of Antibiotics. Mosc. Univ. Biol. Sci. Bull..

[44] Yoon S.J., Park J.E., Yang J.H., Park J.W. (2002). OxyR regulon controls lipid peroxidation-mediated oxidative stress in *Escherichia coli*. J. Biochem. Mol. Biol..

[45] Yang L., Mih N., Anand A., Park J.-O., Tan J., Yurkovich I.T., Monk J.M., Lloyd C.T., Sandberg T.E., Seo S.W. (2019). Cellular responses to reactive oxygen species are predicted from molecular mechanisms. Proc. Natl. Acad. Sci. USA.

[46] Hong Y., Zeng J., Wang X., Drlica K., Zhao X. (2019). Post-stress bacterial cell death mediated by reactive oxygen species. Proc. Natl. Acad. Sci. USA.

[47] Paulander W., Wang Y., Folkesson A., Charbon G., Løbner-Olesen A., Ingmer H. (2014). Bactericidal antibiotics increase hydroxyphenyl fluorescein signal by altering cell morphology. PLoS ONE.

[48] Yeom J., Imlay J.A., Park W. (2010). Iron homeostasis affects antibiotic-mediated cell death in *Pseudomonas* species. J. Biol. Chem..

[49] Arriaga-Alba M., Rivera-Sánchez R., Parra-Cervantes G., Barro-Moreno F., Flores-Paz R., García-Jeménez E. (2000). Antimutagenesis of beta-carotene to mutations induced by quinolone on *Salmonella typhimurium*. Arch. Med. Res..

[50] Kohanski M.A., Dwyer D.J., Wierzbowski J., Cottarel G., Collins J.J. (2008). Mistranslation of membrane proteins and two-component system activation trigger antibiotic-mediated cell death. Cell.

[51] Kolodkin-Gal I., Sat B., Keshet A., Engelberg-Kulka H. (2008). The communication factor EDF and the toxin-antitoxin module mazEF determine the mode of action of antibiotics. PLoS Biol..

[52] Couturier M., Bahassi M., Van Melderen L. (1998). Bacterial death by DNA gyrase poisoning. Trends Microbiol..

[53] Foti J.J., Devadoss B., Winkler J.A., Collins J.J., Walker G.C. (2012). Oxidation of the guanine nucleotide pool underlies cell death by bactericidal antibiotics. Science.

[54] Dorsey-Oresto A., Lu T., Mosel M., Wang X., Salz T., Drlica K., Zhao X. (2013). YihE kinase is a central regulator of programmed cell death in bacteria. Cell Rep..

[55] Ezraty B., Vergnes A., Banzhaf M., Duverger Y., Huguenot A., Brochado A.R., Su S.-Y., Espinosa L., Loiseau L., Py B. (2013). Fe-S cluster biosynthesis controls uptake of aminoglycosides in a ROS-less death pathway. Science.

[56] Barber A.E., Norton J.P., Spivak A.M., Mulvey M.A. (2013). Urinary tract infections: Current and emerging management strategies. Clin. Infect. Dis..

[57] Chua N.G., Zhou Y.P., Tan T.T., Lingegowda P.B., Lee W., Lim T.P., Teo J., Cai Y., Kwa A.L. (2015). Polymyxin B with dual carbapenem combination therapy against carbapnemase-producing *Klebsiella pneumonia*. J. Infect..

[58] Sampson T.R., Liu X., Schroeder M.R., Kraft C.S., Burd E.M., Weiss D.S. (2012). Rapid killing of Acinetobacter baumannii by polymyxins is mediated by a hydroxyl radical death pathway. Antimicrob. Agents Chemother..

[59] Walling C., King T.E., Mason H.S., Morrison M. (1982). The Nature of the Primary Oxidants in Oxidations Mediated by Metal Ions. Oxidases and Related Redox Systems: Proceedings of the Third International Symposium.

[60] Belenky P., Ye J.D., Porter C.B., Cohen N.R., Lobritz M.A., Ferrante T., Jain S., Korry B.J., Schwarz E.G., Walker G.C. (2015). Bactericidal Antibiotics Induce Toxic Metabolic Perturbations that Lead to Cellular Damage. Cell Rep..

[61] Luan G., Hong Y., Drlica K., Zhao X. (2018). Suppression of Reactive Oxygen Species Accumulation Accounts for Paradoxical Bacterial Survival at High Quinolone Concentration. Antimicrob. Agents Chemother..

[62] Malik M., Hussain S., Drlica K. (2007). Effect of anaerobic growth on quinolone lethality with *Escherichia coli*. Antimicrob. Agents Chemother..

[63] Malik M., Hoatam G., Chavda K., Kerns R.J., Drlica K. (2010). Novel approach for comparing abilities of quinolones to restrict the emergence of resistant mutants during quinolone exposure. Antimicrob. Agents Chemother..

[64] Hong Y., Li L., Luan G., Drlica K., Zhao X. (2017). Contribution of reactive oxygen species to thymineless death in *Escherichia coli*. Nat. Microbiol..

[65] Eghbaliferiz S., Iranshahi M. (2016). Prooxidant activity of polyphenols, flavonoids, anthocyanins and carotenoids: Updated review of mechanisms and catalyzing metals. Pharmacol. Res..

[66] Ajiboye T.O., Habibu R.S., Saidu K., Haliru F.Z., Ajiboye H.O., Aliyu N.O., Ibitoye O.B., Uwazie J.N., Muritala H.F., Bello S.A. (2017). Involvement of oxidative stress in protocatechuic acid-mediated bacterial lethality. Microbiologyopen.

[67] Singh D., Narayanamoorthy S., Gamre S., Majumdar A.G., Goswami M., Gami U., Cherian S., Subramanian M. (2018). Hydroxychavicol, a key ingredient of *Piper betle* induces bacterial cell death by DNA damage and inhibition of cell division. Free. Radic. Biol. Med..

[68] Singh D., Majumdar A.G., Gamre S. (2021). Mahesh Subramanian Membrane damage precedes DNA damage in hydroxychavicol treated *E. coli* cells and facilitates cooperativity with hydrophobic antibiotics. Biochimie.

[69] Sinsinwar S., Vadivel V. (2020). Catechin isolated from cashew nut shell exhibits antibacterial activity against clinical isolates of MRSA through ROS-mediated oxidative stress. Appl. Microbiol. Biotechnol..

[70] Ajiboye T.O., Skiebe E., Wilharm G. (2018). Phenolic acids potentiate colistin-mediated killing of *Acinetobacter baumannii* by inducing redox imbalance. Biomed. Pharmacother..

[71] Xiong L.G., Chen Y.J., Tong J.W., Huang J.A., Li J., Gong Y.S., Liu Z.H. (2017). Tea polyphenol epigallocatechin gallate inhibits *Escherichia coli* by increasing endogenous oxidative stress. Food Chem..

[72] Hussain R.M., Abdullah N.F., Amom Z. (2016). Killing of *Staphylococcus aureus* by allylpyrocatechol is potentiated by induction of intracellular oxidative stress and inhibition of catalase activity. J. Integr. Med..

[73] Volk A.P.D., Moreland J.G. (2014). ROS-Containing Endosomal Compartments: Implications for Signaling. Methods Enzymol..

[74] Cooke J., Dryden M., Patton T., Brennan J., Barrett J. (2015). The antimicrobial activity of prototype modified honeys that generate reactive oxygen species (ROS) hydrogen peroxide. BMC Res. Notes.

[75] Aribisala J.O., Nkosi S., Idowu K., Nurain I.O., Makolomakwa G.M., Shode F.O., Sabiu S. (2021). Astaxanthin-Mediated Bacterial Lethality: Evidence from Oxidative Stress Contribution and Molecular Dynamics Simulation. Oxidative Med. Cell. Longev..

[76] Oliveira J.T.A., Souza P.F.N., Vasconcelos M.I., Dias L.P., Martins T.F., Van Tilburg M.F., Guedes M.I.F., Sousa D.O.B. (2019). Mo-CBP3-PepI, Mo-CBP3-PepII, and Mo-CBP3-PepIII are synthetic antimicrobial peptides active against human pathogens by stimulating ROS generation and increasing plasma membrane permeability. Biochimie.

[77] Ong K.S., Cheow Y.L., Lee S.M. (2017). The role of reactive oxygen species in the antimicrobial activity of pyochelin. J. Adv. Res..

[78] Wang L., Monro S., Cui P., Yin H., Liu B., Cameron C.G., Xu W., Hetu M., Fuller A., Kilina S.V. (2019). Heteroleptic Ir (III)N6 complexes with long-lived triplet excited states and in vitro photobiological activities. ACS Appl. Mater. Interfaces.

[79] Wang Z., Bai H., Lu C., Hou C., Qiu Y., Zhang P., Duan J., Mu H. (2019). Light controllable chitosan micelles with ROS generation and essential oil release for the treatment of bacterial biofilm. Carbohydr. Polym..

[80] Song H., Wang Y., Wu J., Gu S., Li H. (2018). Fabrication of Bis-Quaternary Ammonium Salt as an Efficient Bactericidal Weapon Against *Escherichia coli* and *Staphylococcus aureus*. ACS Omega.

[81] Hu L., Wang H., Xia T., Fang B., Shen Y., Zhang Q., Tian X., Zhou H., Wu J., Tian Y. (2018). Two-Photon-Active Organotin (IV) Complexes for Antibacterial Function and Superresolution Bacteria Imaging. Inorg. Chem..

[82] Liu H., Qu X., Kim E., Lei M., Dai K., Tan X., Xu M., Li J., Liu Y., Shi X. (2018). Bio inspired redox-cycling antimicrobial film for sustained generation of reactive oxygen species. Biomaterials.

[83] Lee W., Woo E.R., Lee D.G. (2019). Effect of apigenin isolated from *Aster yomena* against *Candida albicans*: Apigenin-triggered apoptotic pathway regulated by mitochondrial calcium signaling. J. Ethnopharmacol..

[84] Abadi P.G., Shirazi F.H., Joshaghani M., Moghimi H.R. (2018). Influence of formulation of ZnO nanoblokes containing metallic ions dopants on their cytotoxicity and protective factors: An in vitro study on human skin cells exposed to UVA radiation. Toxicol. Rep..

[85] Ali D., Tripathi A., Al Ali H., Shahi Y., Mishra K.K., Alarifi S., Alkahtane A.A., Manohardas S. (2018). ROS dependent Bax/Bcl2 and caspase 3 pathway-mediated apoptosis induced by zineb in human keratinocyte cells. OncoTargets Ther..

[86] Piao M.J., Ahn M.J., Kang K.A., Ryu Y.S., Hyun Y.J., Shilnikova K., Zhen A.X., Jeong J.W., Choi Y.H., Kang H.K. (2018). Particulate matter 2.5 damages skin cells by inducing oxidative stress, subcellular organelle dysfunction, and apoptosis. Arch. Toxicol..

[87] Jin S.P., Li Z., Choi E.K., Lee S., Kim Y.K., Seo E.Y., Chung J.H., Cho S. (2018). Urban particulate 859 matter in air pollution penetrates the barrier-disrupted skin and produces ROS-dependent cutaneous inflammatory response in vivo. J. Dermatol. Sci..

[88] Lipinski B. (2011). Hydroxyl radical and its scavengers in health and disease. Oxidative Med. Cell. Longev..

[89] Gao F., Shao T., Yu Y., Xiong Y., Yang L. (2021). Surface-bound reactive oxygen species generating nanozymes for selective antibacterial action. Nat. Commun..

[90] Kim S., Woo E.R., Lee D.G. (2019). Synergistic Antifungal Activity of Isoquercitrin: Apoptosis and Membrane Permeabilization Related to Reactive Oxygen Species in *Candida albicans*. IUBMB Life.

[91] Ye J.Z., Su Y.B., Lin X.M., Lai S.S., Li W.X., Ali F., Zheng J., Peng B. (2018). Alanine Enhances Aminoglycosides- Induced ROS Production as Revealed by Proteomic Analysis. Front. Microbiol..

